# PTEN loss correlates with T cell exclusion across human cancers

**DOI:** 10.1186/s12885-021-08114-x

**Published:** 2021-04-19

**Authors:** Ziying Lin, Lixia Huang, Shao Li Li, Jincui Gu, Xiaoxian Cui, Yanbin Zhou

**Affiliations:** 1 Department of Respiratory and Critical Care Medicine, The First Affiliated Hospital of Sun Yat-sen University, Guangzhou, China; 2 Department of Respiratory Medicine, The 8th Affiliated Hospital of Sun Yat-sen University, Shenzhen, China

**Keywords:** PTEN, PI3K signaling, Tumor immune microenvironment, T cells, Immunotherapy

## Abstract

**Background:**

Recent evidences had shown that loss in phosphatase and tensin homolog deleted on chromosome 10 (PTEN) was associated with immunotherapy resistance, which may be attributed to the non-T-cell-inflamed tumor microenvironment. The impact of PTEN loss on tumor microenvironment, especially regarding T cell infiltration across tumor types is not well understood.

**Methods:**

Utilizing The Cancer Genome Atlas (TCGA) and publicly available dataset of immunotherapy, we explored the correlation of PTEN expressing level or genomic loss with tumor immune microenvironment and response to immunotherapy. We further investigated the involvement of PI3K-AKT-mTOR pathway activation, which is known to be the subsequent effect of PTEN loss, in the immune microenvironment modulation.

**Results:**

We reveal that PTEN mRNA expression is significantly positively correlated with CD4/CD8A gene expression and T cells infiltration especially T helpers cells, central memory T cell and effector memory T cells in multiples tumor types. Genomic loss of PTEN is associated with reduced CD8+ T cells, type 1 T helper cells, and increased type 2 T helper cells, immunosuppressed genes (e.g. VEGFA) expression. Furthermore, T cell exclusive phenotype is also observed in tumor with PI3K pathway activation or genomic gain in PIK3CA or PIK3CB. PTEN loss and PI3K pathway activation correlate with immunosuppressive microenvironment, especially in terms of T cell exclusion. PTEN loss predict poor therapeutic response and worse survival outcome in patients receiving immunotherapy.

**Conclusion:**

These data brings insight into the role of PTEN loss in T cell exclusion and immunotherapy resistance, and inspires further research on immune modulating strategy to augment immunotherapy.

**Supplementary Information:**

The online version contains supplementary material available at 10.1186/s12885-021-08114-x.

## Background

Phosphatase and tensin homolog deleted on chromosome 10 (PTEN) is well known as the tumor suppressor gene for its negative regulation on phosphatidylinositol 3-kinase (PI3K) pathway [1]. Activated PI3K can convert phosphatidylinositol (4,5) biphosphate (PIP2) into phosphatidylinositol (3,4,5) triphosphate (PIP3), which subsequently activates AKT/mTOR pathway and drives tumor proliferation and progression [2]. PTEN exerts its tumor suppressive function by dephosphorylating PIP3 and thus prevent the activation of PI3K-AKT-mTOR pathway [3]. Other than the negative regulation of oncogene pathway, emerging evidences suggested that PTEN may also play a role in immune modulation and interfering with the therapeutic efficacy of Immunotherapy [4–9].

Immunotherapy that modulate anti-tumor immune response by blocking immune checkpoint is the landmark victory in the treatment of advanced cancer, as accumulating evidences have proven the durable response in multiple tumor types [10]. Marked improvement in clinical outcome had been achieved by checkpoint blockers targeting programmed death-1/programmed death ligand-1 (PD-1/PD-L1) in a wide spectrum of cancer entities including non-small cell lung cancer (NSCLC), melanoma etc. [11, 12]. Yet despite the promising clinical benefit and the broad activity, the remaining downside for immunotherapy is that only a restricted percentage of patients response to the therapy [13]. That is, intrinsic resistance is widely exist among patients, though the mechanism of which is not clearly understood. Previous studies tried to approach this issue from the perspective of tumor microenvironment (TME) and found that non-T-cell-inflamed tumor microenvironment was closely associate with poor therapeutic response to immunotherapy, particularly with anti-PD-1 antibodies [14, 15]. Great effort is still ongoing to decipher the underlying genomic or molecular mechanism for immune exclusion [16–18].

Among the newly proposed molecular alteration that disfavor T-cell infiltration and contribute to immune evasion, PTEN loss has gain more and more attention as multiple studies have suggested its involvement in immunotherapy resistance. Weiyi Peng etc. first proved that loss of PTEN was associated with reduced number and impaired function of tumor-infiltrating T cells, and poor response to anti-PD-1 treatment in melanoma patients [9]. Subsequent studies also reported similar finding for other tumor types including NSCLC, triple-negative breast cancer, prostate cancer, glioblastoma, Uterine Leiomyosarcoma etc. [4–8]. With these well-recognized phenomena, emerging studies tried to tackle the specific mechanism of T cell exclusion mediated by PTEN, and found that PTEN loss was associated with increased expression of certain immune suppressive genes like forkhead box P3 (FOXP3), IDO1, VEGFA etc. [19]. But still, the downstream molecular pathway by which PTEN mediate tumor immune microenvironment remains a mystery. There is evidence suggesting that PI3K pathway activation after PTEN loss may contribute to T cell exclusion, as PI3K targeting drugs can synergize with immunotherapy in the treatment of melanoma with PTEN loss [9, 20]. Whether PTEN loss in tumor cause immune exclusion by way of PI3K-AKT-mTOR pathway is yet to be confirmed by further solid evidence.

To investigate the influence of PTEN loss on immune microenvironment across solid tumors, and to decide the involvement of PI3K-AKT-mTOR pathway amidst, we performed an integrative analysis of The Cancer Genome Atlas (TCGA) to clarify the correlation of immune cells infiltration with PTEN loss as well as PI3K-AKT-mTOR pathway activation. We found that both PTEN loss and activation of PI3K pathway were associated with reduced T cell infiltration and enhanced immune suppressive status in multiple tumor types. The correlation of PTEN loss with poor response to immunotherapy was also verified with publicly available data from immunotherapy trials. Our findings bring insight into the novel immunotherapy resistance mechanism associated with PTEN loss, and convey implication for improving immunotherapy efficacy.

## Methods

### TCGA cancer database

RNA-seq data of 21 solid tumor types from The Cancer Genome Atlas (TCGA) were obtained from GEO database (GEO: GSE62944), where raw data of all the tumor types were reprocessed by aligning the fastq files downloaded from the Cancer Genomics Hub so that the expression value of the genes could be compared between the different samples [21]. Mutation data and copy number variants of PTEN, PIK3CA and PIK3CB were obtained from cBioportal database (https://www.cbioportal.org/). The GISTIC2.0 [22] annotation of CNAs had been previously binned into − 2, − 1, 0, 1, and 2, representing total copy loss, hemizygous deletion, euploidy, copy number gain, and high fold amplification. PTEN with loss-of-function mutation or with copy number < − 1 were defined as PTEN loss, otherwise defined as PTEN intact. PIK3CA with gain-of-function mutation or with copy number > 1 were defined as PIK3CA gain, otherwise defined as wide-type PIK3CA. The same rules were also applied for PIK3CB to categorize PIK3CB gain and wide-type PIK3CB. Level 3 reverse phase protein array (RPPA) antibody-level protein abundance data (release date January 28, 2016; patch July 14, 2016) produced by The University of Texas MD Anderson Cancer Center were downloaded from TCPA database (https://tcpaportal.org/tcpa/my_protein.html), which mainly consists of TCGA tumor tissue sample sets. Phosphorylation level of AKT, mTOR, STAT3 estimated using median-centered normalized values corresponding to antibody Phospho-Akt (Ser473), Phospho-Akt (Thr308), Phospho-mTOR (Ser2448), Phospho-STAT3 (Tyr705) were obtained from the database. Only part of the included cases have TCPA data available. All the basic information of 21 solid tumors included in the current study were summarized in supplementary Table 1.

### Estimation of immune cell enrichment from RNA-seq data

xCell is a webtool that performs cell type enrichment analysis for different immune and stroma cell types based on the bulk-tissue RNA-seq data. The enrichment abundance of 24 immune cell types in tumor microenvironment was estimated by xCell using Bindea signatures (https://xcell.ucsf.edu/). All TCGA tumors were processed by xCell to generate enrichment scores for each immune cell type across all samples by integrating single-sample gene set enrichment analysis (ssGSEA) and deconvolution methods [23].

### Pathway analysis

Differential expression of all the genes between tumors with intact PTEN and tumors with PTEN loss in each cancer types were evaluated using DESeq2 package (72) in R software environment. Genes were first ranked according to log2(fold change) and then applied to GSEA analysis using GSEAPreranked tool (GSEA 4.0.3) with a T-cell inflamed signature derived from published literature [24]. Enrichment score of certain pathway activation e.g. PI3K/AKT pathway etc. were computed for each individual tumor based on the normalized transcriptome data of Human gene sets for PI3K/AKT pathway (Reactome_PIP3_activates_AKT_signaling) were obtain from in Molecular Signatures Database v7.0 (MsigDB7.0). Enrichment score was generated by single-sample gene set enrichment analysis (ssGSEA) using R-package (GSEABase and GSVA).

### Data obtaining from immunotherapy clinical trial

In order to verify the correlation between PTEN loss and clinical response to immunotherapy, published data of immunotherapy clinical trials (Chen et al., Cancer Discovery 2016 [25] and Riaz et al., Cell 2017 [26]) with publicly available clinical and transcriptome data were obtained and reanalyzed in the present study. In the Chen et al. cohort, patients with metastatic melanoma were initially treated with CTLA4 blockade and were then treated with PD-1 blockade if they did not respond or progressed on CTLA4 blockade. Transcriptome expression of PTEN, CD4, CD8A, VEGFA and clinical data of Chen et al. cohort were retrieved from the supplementary materials of the published study. In the Riaz et al. cohort, a total of 65 patients with advanced melanoma who haven’t received any immunotherapy (ipilimumab-naïve) or had progressed on ipilimumab (ipilimumab-progressed) were recruited and treated with PD-1 blockade (Nivolumab). As for Riaz et al. cohort, we only analyzed the transcriptome data derived from specimen biopsied prior to anti-PD-1 treatment. Transcriptome data of Riaz et al. cohort were downloaded from GEO database (GSE91061), while clinical information were obtained from the supplementary material of the corresponding published study. Detailed information of this two datasets were shown in supplementary Table 2–3.

### Statistical analysis

**Fig. 1:**
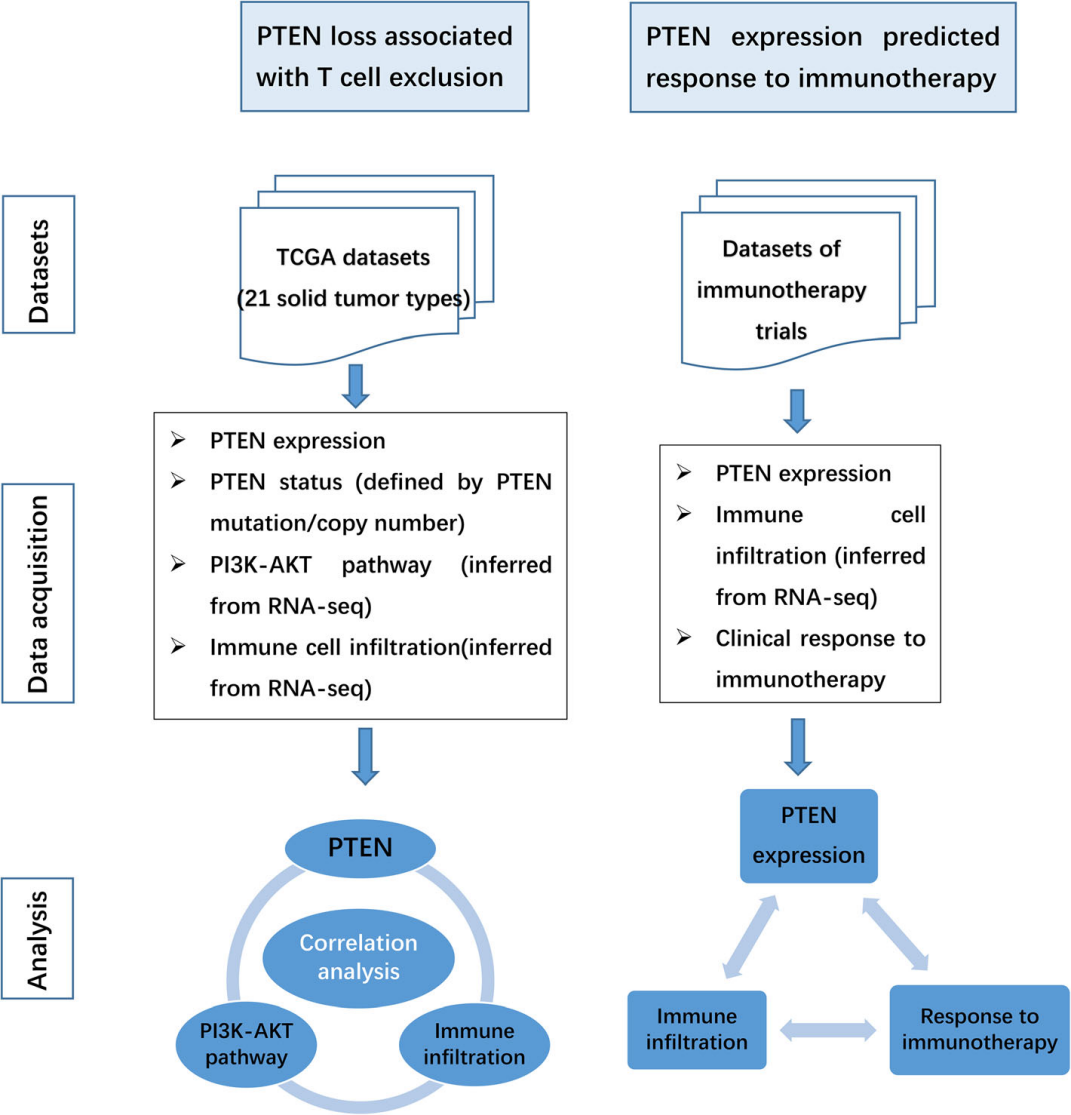
Flow scheme of the analysis process

The flow scheme of the whole analysis process was demonstrated in Fig. 1. Assessments of difference in continuous variables between two groups were decided by two-sample t test. Pearson correlation analysis was carried out to determine the correlation between two continuous variables. Logarithmic transformation was carried out for single gene expression to obtain variables that comply normal distribution and suitable for t test or correlation analysis. T test comparison was omitted for certain tumor types when the observed sample size was too small in one group. The prognostic significance of categorical variables was estimated using Kaplan–Meier plots (log-rank test). All statistical analyses and data presentations were performed in R language 3.4.1 (http://www.r-project.org).

## Results

### PTEN expression is positively correlated with T cell infiltration

**Fig. 2:**
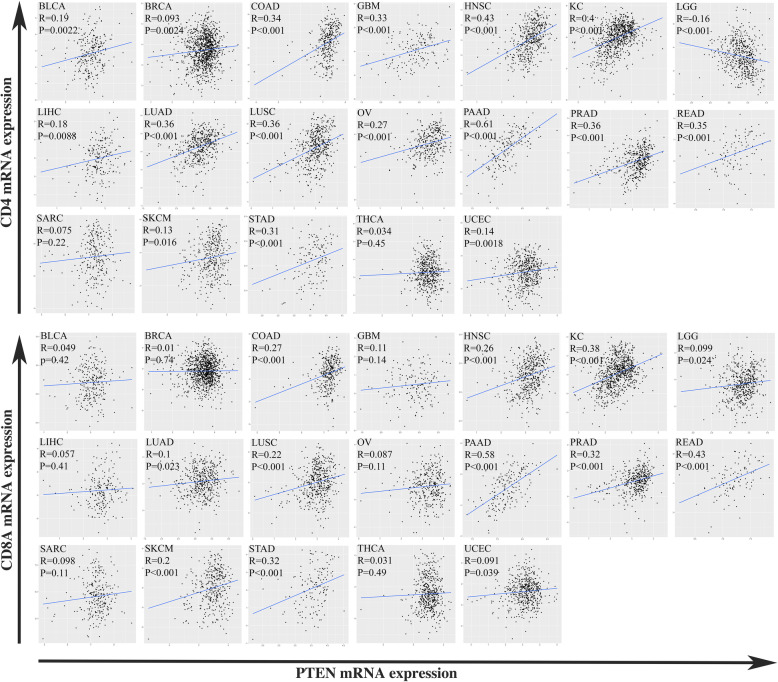
Positive correlation between expression of PTEN and T-cell-inflamed genes. Dot plots of PTEN mRNA expression (log transferred) on x-axis and T-cell-inflamed genes (CD4, CD8A) mRNA expression (log transferred) on y-axis per tumor types. Correlation coefficients (*R* value) and *P* value of Pearson Correlation were shown for each tumor type. mRNA expression of PTEN was positively correlated with expression level of CD4 and CD8 in multiple tumor types. Abbreviations: BLCA, Bladder Urothelial Carcinoma; BRCA, Breast invasive carcinoma; COAD, Colon adenocarcinoma; GBM, Glioblastoma multiforme; HNSC, Head and Neck squamous cell carcinoma; KICH, Kidney Chromophobe; KIRC, Kidney renal clear cell carcinoma; KIRP, Kidney renal papillary cell carcinoma; LGG, Brain Lower Grade Glioma; LIHC, Liver hepatocellular carcinoma; LUAD, Lung adenocarcinoma; LUSC, Lung squamous cell carcinoma; OV, Ovarian serous cystadenocarcinoma; PAAD, Pancreatic adenocarcinoma; PRAD, Prostate adenocarcinoma; READ, Rectum adenocarcinoma; SARC, Sarcoma; SKCM, Skin Cutaneous Melanoma; STAD, Stomach adenocarcinoma; THCA, Thyroid carcinoma; UCEC, Uterine Corpus Endometrial Carcinoma

To investigate the impact of PTEN expression on T cell infiltration, the correlation between mRNA expression of PTEN and T cell specific genes (CD4, CD8A) in different solid tumor were determined by Pearson correlation analysis. As demonstrated in Fig. 2, significant positive correlation between gene expression of PTEN and CD4 was observed in most of the tumor types except for low-grade glioma (LGG), sarcoma (SARC) and thyroid carcinoma (THCA). Similar relationship was also observed between gene expression of PTEN and CD8A, though the correlation was less significant as that between PTEN and CD4.

**Fig. 3:**
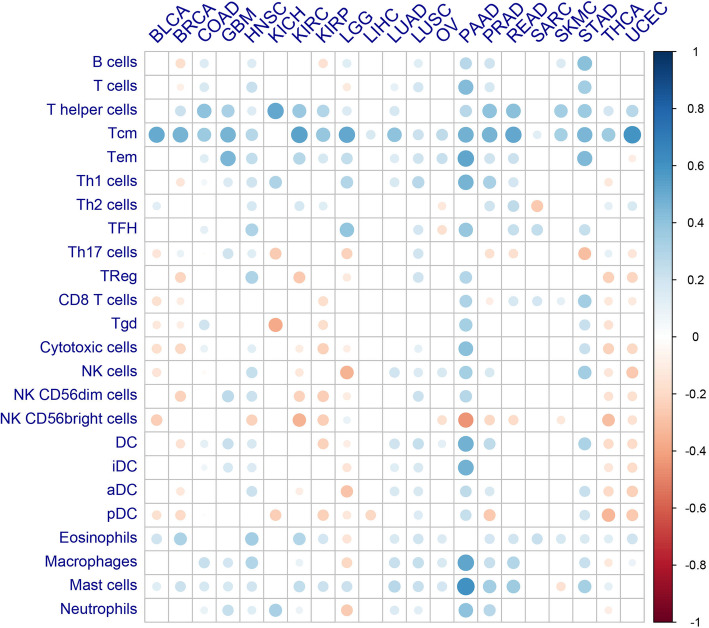
The Correlation between the Expression of PTEN and the Infiltration of Immune Cells. The Correlation between the mRNA Expression of PTEN and 24 types of infiltrating immune cells was evaluated by Pearson Correlation analysis. Node color is determined by correlation, and node size indicates the significance of correlation. Only nodes with Correlation significance (*P* value) < 0.05 were shown. PTEN expression was positively correlated with T cells, T helper cells, memory T cells in multiple cancer types. Abbreviations: Th1, type 1 T helper cells; Th2, type 2 T helper cells; Tem, effector memory T cells; Tcm, central memory T cells; TFH, follicular T helper cells; TReg, regulatory T cells; Tgd, γδ T cells; NK cells, nature killer cells; DC, dendritic cells; aDC, activated dendritic cell; pDC, plasmacytoid dendritic cell; iDC, immature dendritic cell; full terms of cancer abbreviation were shown in Fig. 2

To further evaluate the impact of PTEN expression on the infiltration of different subpopulations of tumor-infiltrated leukocytes (TILs), we obtain the infiltrating abundance of 24 immune cells, including 13 adaptive immune cells and 11 innate immune cells. The correlation between PTEN mRNA expression and different immune cells infiltrating abundance was determined by Pearson correlation analysis, the yielded results were shown in a dot plot, where the node size indicates the significance of correlation significance and the color denotes the degree of correlation. As shown in Fig. 3, the mRNA expression of PTEN was positive correlated with T helper cells, central memory T cells and effector memory T cells in most of the solid tumors. Specifically, PTEN expression was positively correlated with type 1 T helper (Th1) cells but not type 2 T helper (Th2) cells. It’s worth mentioned that no significant positive correlation was observed between PTEN expression and CD8 T cells or cytotoxic cell for most tumor types in the correlation analysis.

### PTEN loss is associated with immunosuppressive microenvironment in pan-cancer

**Fig. 4:**
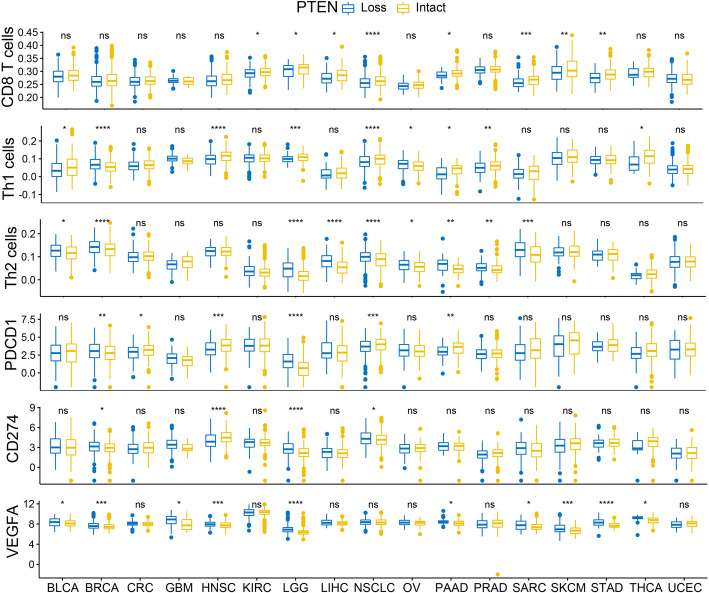
Difference of T cells infiltration or immunosuppressive markers between tumors with PTEN loss and tumors with Intact PTEN. T cells (CD8+ T cells, Th1 cells, Th2 cells) infiltration level and mRNA Expression of Immunosuppressive markers (PD-L1, encoded by CD274; PD-1, encoded by PDCD1; VEGF, encoded by VEGFA) between tumor with PTEN loss or Intact PTEN. *p < 0.05; **p < 0.01; ***p < 0.001; ****p < 0.0001; ns, not significant. Tumors with PTEN loss demonstrated significant lower infiltrating level of CD8 T cell and type 1 T helper cells (Th1 cells) and expression level, but increased infiltration of type 2 T helper cells (Th2 cells) and expression of VEGFA in multiple cancer types. No general pattern was observed for the correlation of PTEN status with expression level of PDCD1 and CD274 among different cancer types. Abbreviations: full terms of cancer abbreviation were shown in Fig. 2

Except for expression level, we want to see whether genomic alteration of PTEN also has an impact on the tumor immune microenvironment. We first classified each tumor sample as PTEN loss or PTEN intact based on it genomic alteration status (as detailly described in the method). Expectedly, PTEN loss was associated with significant lower PTEN expression (supplementary Fig 1). We compare the infiltrating abundance of immune cells between tumor with PTEN loss or intact PTEN, and found that PTEN loss was associated with significantly reduced infiltration of CD8 T cell, Th1 cell, and increased infiltration of Th2 cell in multiple tumor types (Fig. 4). To further confirm the relevance of PTEN status in T cell inflamed macroenvironment, GSEA pathway analysis was performed to compared the difference of T cell inflamed pathway between tumors with PTEN loss and tumors with intact PTEN. We found that T cell inflamed signature was highly enriched in tumors with wide-type PTEN in most cancer types (supplementary Fig 2). As for the innate population, dendritic cells (DC), which function as the major antigen presenting cells during T cell activation, was also significantly reduced in multiple tumors with PTEN loss (supplementary Fig. 3A). We also evaluated the association between PTEN loss and the expression of immune suppressive markers that had been previously reported to be associated with PTEN. We did not observed ubiquitous changes in the expression of PDCD1 and CD274 for most tumor types with PTEN loss, with PTEN-loss tumors had elevated PDCD1 and CD274 for some cancer types, but demonstrated downregulated expression in some others and manifested no difference in most cancer types (Fig. 3). Similar findings were also observed for other immune suppressive genes like IDO1, FOXP3 and CCL2, CSF1, IL6, etc. (supplementary Fig 4). However, VEGFA expression was significantly upregulated in tumors with PTEN loss for most of the tumor types, with only a few others demonstrating no significant difference (Fig. 4).

### PI3K-AKT-mTOR pathway might contribute to T cell exclusion associated with PTEN loss

**Fig. 5:**
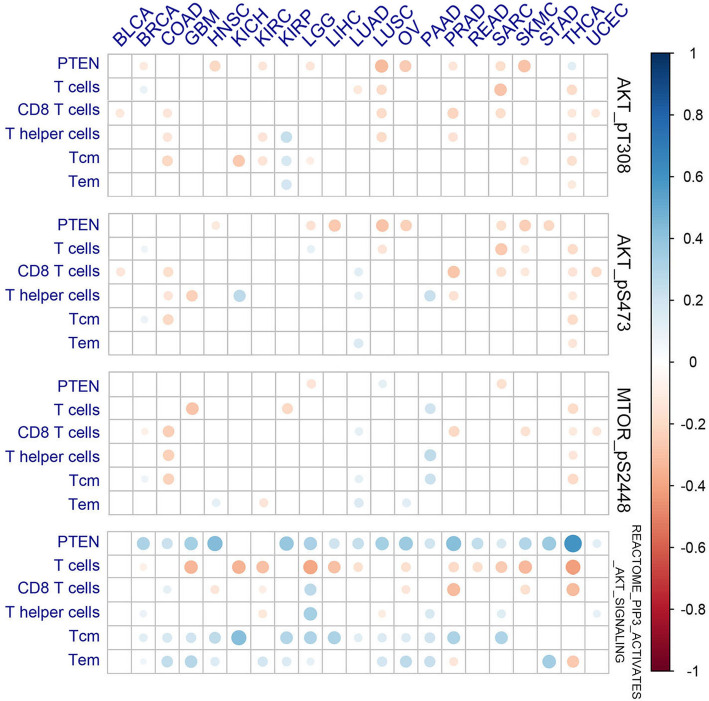
The Correlation of PI3K-AKT-mTOR pathway activation with PTEN expression and the T cells infiltration. The Correlation of PI3K-AKT-mTOR pathway activation (represented by p-AKT [phosphorylated at Thr308 or Ser473], p-mTOR [phosphorylated at Ser2448], and ssGSEA score of PI3K pathway) with PTEN expression and the T cells infiltration was evaluated by Pearson Correlation analysis. Node color is determined by correlation, and node size indicates the significance of correlation. Only nodes with Correlation significance (*P* value) < 0.05 were shown. Abbreviations: Tem, effector memory T cells; Tcm, central memory T cells; full terms of cancer abbreviation were shown in Fig. 2

It’s well known that PTEN loss is associated with activation of PI3K-AKT-mTOR pathway, which had also been linked with immunosuppressive microenvironment and resistance to immunotherapy [9, 27]. Here we further explored the impact of PI3K-AKT-mTOR pathway on tumor immune microenvironment and whether it’s the downstream mechanism by which PTEN mediates immune changes in tumor. The activation of PI3K-AKT-mTOR pathway was quantified by ssGSEA score of PI3K pathway geneset (REACTOME_PIP3_ACTIVATES_AKT_SIGNALING), expression level of phosphorylated protein (p-AKT [pT308], p-AKT [pS473] and p-mTOR [pS2448]) respectively. We first determined the correlation between PI3K-AKT-mTOR pathway activation and PTEN loss, and found that PTEN loss was associated with significantly increased p-AKT (pT308, pS473) in multiple tumor types, but no changes in p-mTOR (pS2448) for most tumor types. Unexpectedly, PI3K pathway scoring was even reduced in tumors with PTEN loss for multiple tumor types (supplementary Fig. 1). The correlation between PI3K-AKT-mTOR pathway activation and immune cell infiltration as well as PTEN expression was determined by Pearson correlation analysis. As shown in Fig. 5, phosphorylation level of AKT (pT308 and pS473) was negatively associated with PTEN expression and T cell infiltration only in several tumor types. No specific correlation with T cell infiltration or PTEN expression was observed for p-mTOR in most tumors. PI3K pathway scoring was negatively associated T cell infiltration but positively correlation with PTEN expression at the same time. PI3K pathway scoring was also positively correlated with memory T cell infiltration, which can owe to its positive correlation with PTEN expression level.

**Fig. 6:**
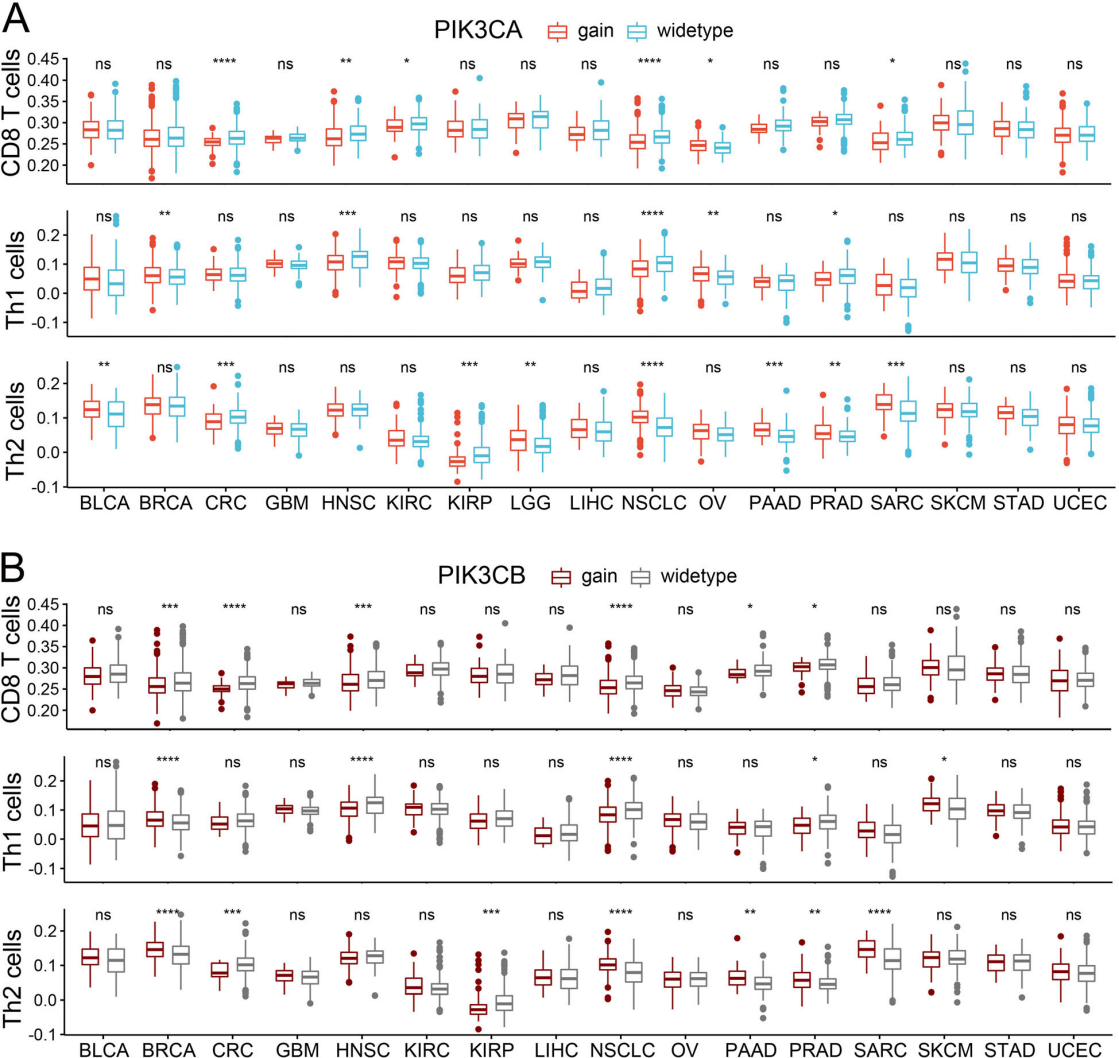
Difference of T cells infiltration between tumors with genomic gain in PIK3CA/PIK3CB and tumors with wide-type PIK3CA/PIK3CB. **a** Difference of T cells infiltration between tumors with PIK3CA gain and tumors with wide-type PIK3CA; **b** Difference of T cells infiltration between tumors with PIK3CB gain and tumors with wide-type PIK3CB. *p < 0.05; **p < 0.01; ***p < 0.001; ****p < 0.0001; ns, not significant. Genomic gain in PIK3CA/PIK3CB were associated with reduced infiltration of CD8 T cell and type 1 T helper cells (Th1 cells), but increased infiltration of type 2 T helper cells (Th2 cells) in multiple cancer types. Abbreviations: full terms of cancer abbreviation were shown in Fig. 2

As expression level of p-AKT, p-mTOR or PI3K pathway scoring were not derived from pure tumor mass, but also included those of the infiltrating immune population and other stromal cells. We further focus on the genomic alteration of PI3K, which were more exclusively occur to malignant cells. PIK3CA and PIK3CB are the oncogenic genes that encode the subunits of PI3K, P110α and P110β respectively, the genomic alteration (gain-of-function mutation or copy number amplification) of which had been associated with the development and progression of multiple malignant disease [28, 29]. To further verified the involvement of PI3K pathway activation in tumor immune modulation, association between T cells infiltration and genomic alteration of PIK3CA or PIK3CB was evaluated. Intriguingly, genomic gain in PIK3CA or PIK3CB were associated decreased CD8 T cells, Th1 cell but increased Th2 cells infiltration in multiple tumor types (Fig. 6), which was similar to what we observed for PTEN loss.

### PTEN expression predict response to immunotherapy

**Fig. 7:**
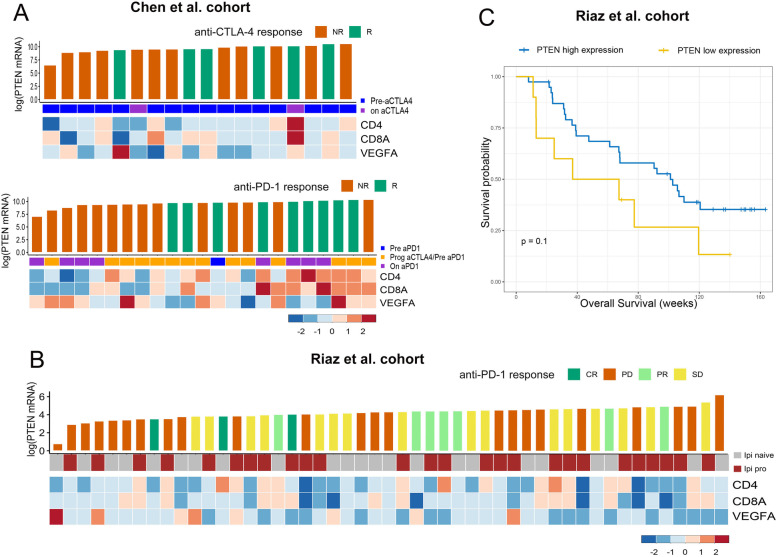
Predictive value of PTEN expression in patients receiving immunotherapy. **a** Bar plot and heatmap demonstrated the mRNA expression (log transferred) level of PTEN and immune related genes (CD4, CD8A, VEGFA) respectively for patients receiving anti-CTLA4 blockade or anti-PD-1 blockade in Chen et al. cohort, with the samples ranked in the ascending order according to PTEN expression. Response to immunotherapy (R: response; NR: not response) was denoted by the color of the bar. Patients with higher PTEN expression demonstrated higher expression of T infiltration markers (CD4, CD8) and better response to CTLA-4 blockade or PD-1 blockade in Chen et al. cohort. **b** Bar plot and heatmap demonstrated the mRNA expression (log transferred) level of PTEN and immune related genes (CD4, CD8A, VEGFA) respectively for patients receiving anti-PD-1 treatment in Riaz et al. cohort, with the samples ranked in the ascending order according to PTEN expression. Response to immunotherapy (CR: complete response; PR: partial response; SD: stable disease; PD: progressed diseased) was denoted by the color of the bar. Patients with higher PTEN expression demonstrated higher expression of T infiltration markers (CD4, CD8) and better response PD-1 blockade in Riaz et al. cohort. **c** Kaplan-Meier plots of overall survival difference between patients with low PTEN expression (the bottom fifth, *n* = 10) and high PTEN expression (the top four fifths, *n* = 39) in Riaz et al. cohort. Patients with higher PTEN expression demonstrated improved overall survival after anti-PD-1 immunotherapy in Riaz et al. cohort

Prognostic impact of PTEN in patients underwent surgical treatment was evaluated based on TCGA database, where no survival difference was observed across most tumor types (supplementary Fig 5). To further verified the significance of PTEN in immunotherapy, the correlation between PTEN expression and response to check point blockade was further evaluated in two independent cohort (Chen et al. cohort and Riaz et al. cohort). For patients with advanced melanoma receiving anti-CTLA-4 treatment (*n* = 18) in Chen et al. cohort, cases with low mRNA PTEN expression responded poorly to anti-CTLA4 blockade (Fig. 7a). Similarly, for patients receiving anti-PD-1 treatment in Chen et al. cohort (*n* = 23) (Fig. 7a) and Riaz et al. cohort (*n* = 49) (Fig. 7b), non-responders were predominately among those with low mRNA PTEN expression. Of note, tumors with low PTEN expression also demonstrate low CD4/CD8A mRNA expression and high VEGFA mRNA expression at the same time, which is consistent with the findings in TCGA cohort.

We further explored the prognostic significance of PTEN in patients receiving immunotherapy. For patients with advanced melanoma receiving anti-PD-1 treatment (*n* = 49) in Riaz et al. cohort, overall survival were compared between patients with low PTEN expression (the bottom fifth, *n* = 10) and high PTEN expression (the top four fifths, *n* = 39). Patients with low PTEN expression demonstrated worse overall survival (*P* = 0.1) (Fig. 7c).

## Discussion

Defined by lack of T cell infiltration within tumor, non-T-cell-inflamed microenvironment, was one of the major cause for immunotherapy resistance. Our study collected evidence that PTEN loss was one of the tumor-intrinsic mechanism contributing to an immune exclusion phenotype. We profiled the correlation of PTEN loss with T-cell-exclusion microenvironment across different solid tumors based on TCGA database, and found that both genomic loss (loss-of-function mutation or copy number deletion) and low expression of PTEN were associated with immune exclusion in a pan-cancer setting. PTEN’s impact on tumor immune microenvironment might be mediated by PI3K pathway, whose activation was also associated immunosuppressive phenotype in multiple solid tumors. These findings not only facilitate our understanding in the correlation between PTEN loss and immunotherapy resistance, but also inspire continued pursuit on immune modulating strategy to augment immunotherapy.

In this study, we evaluated the correlation of genomic alteration and expression of PTEN with tumor T cells infiltration. Consistent with previous findings, we found both genomic loss or reduced expression of PTEN were associated with decreased T cell trafficking and immune suppressive microenvironment in board range of malignancies [8, 9, 19, 30]. What differs our findings from the published studies is that we first reported T cell infiltration level was positively correlated with PTEN expression level. Also, reduced expression in PTEN predicted poor response to immunotherapy and worse outcome. These findings indicated that PTEN’s impact on immune microenvironment is expression dependent. A recent published study indicated that overexpression of PTEN in tumor cells can enhance T-cell mediated tumor clearance, which also proved that it’s the expression level rather than functional status of PTEN that matters [31]. Another interesting finding in the present study is the strong correlation between PTEN expression and memory T cells (central memory T cells and effector memory T cells). Studies on TME had revealed that memory T cells were mainly located in tertiary lymphoid structures (TLS), which encompass abundant immature T cells, B cells and play an important role in tumor cell clearance [32]. The presence of TLS is not only associated with improved clinical outcome in multiple cancers, but also found to promote immunotherapy response in patients with melanoma or renal cell carcinoma [33, 34]. It’s possible that the involvement of PTEN loss in immunotherapy resistance is partial mediated by TLS. If it’s the case, it can explain the finding reported in a recent study that tumors with wide type PTEN manifested significant higher effector T cells as compared to tumors with mutant PTEN only after the treatment of immunotherapy, when memory T cells will be activated and differentiate into functional T cells [7].

Of note, the analysis of PTEN’s correlation with immune infiltration from the perspective of expression level and genomic alteration status didn’t yield exactly concordant results. For example, we didn’t find significant correlation of PTEN expression with the infiltrating level of CD8 T cell and Th2 cell, which though, were significant reduced and increased respectively in multiple tumors with genomic PTEN loss. Also, memory T infiltration was strongly correlated with PTEN expression, but only demonstrated significant reduction in selected tumor types with PTEN loss. These discrepancy may be attributed to the miscellaneous signal of PTEN that derived from stromal cells in addition to cancer cells. When immune cells or other stromal cell would also contribute to part of the expression level of PTEN, genomic data of PTEN (mutation and copy number variants) were more likely restricted to the tumor cells. Therefore, although we observed a positive correlation between PTEN expression and memory T cells infiltration, we can’t rule out the possibility that correlation is partially attributed to the high expression of PTEN in certain immature T cells. Another explanation for this discordance could be the way we defined loss-of-function mutation for PTEN. Loss-of-function mutations include mutations that cause inhibition of PTEN catalytic activity or mutations that lead to unstable truncated proteins. As the immune modulating function of PTEN might be independent of its catalytic activity, loss-of-function mutation is not necessary synonymous to loss of immune modulating function [35].

Existing study had shown that PTEN loss in tumor cells were associated with increased expression of immunosuppressive cytokines, especially VEGF, which reduce T trafficking as well as its cytotoxic function in TME [9]. Loss of PTEN in glioma was also reported to be associated with increased PD-L1 expression and immune escape [36]. There is still evidence indicates the correlation between PTEN loss on tumor cells and elevation of immunosuppressive markers like IDO1, FOXP3, CCL2, CSF1 ect [37, 38].. Consistent with the published findings, we also found that PTEN loss is associated with increased mRNA expression of VEGFA, IDO1, IL6, CCL2, CSF1 in a wide spectrum of solid tumors. The expression level of PD-1, PD-L1 and FOXP3 were not altered by PTEN loss in most tumors, or even demonstrated reduced expression in some tumors, which might be attributed to the reduced T cells abundance associated with PTEN loss.

Our findings confirmed that PTEN loss in tumor cells was associated with T cell exclusion and an immunosuppressive microenvironment in solid tumors, though the underlying mechanism of which remains a puzzle. As we all know, the cornerstone discovery of PTEN’s biology was the negative regulation of the pro-oncogenic PI3K-AKT-mTOR pathway mediated by dephosphorylation of substrate PIP_3_ [2, 27, 39, 40]. Quite a few studies had tried to tackle the immune modulating mechanism of PTEN from the perspective of PI3K-AKT-mTOR pathway alteration. James S. Waldron etc. found that PTEN loss associated with activation of the PI3K-Akt-mTOR pathway and led to a autologous T-cell apoptosis in glioblastoma, which can be diminished by treatment with inhibitors of PI3K-Akt-mTOR pathway [41]. Another study also indicated that PTEN-depleting melanoma cells promoted the expression of immunosuppressive cytokines in a PI3K-dependent manner [37]. We do observed a negative correlation of p-AKT level and PI3K pathway scoring with T cell infiltration in multiple tumor types, though the correlation is not as strong as we expected. It’s worth mentioned that the data regarding p-AKT level and PI3K pathway scoring were derived from tumor cells as well as the infiltrating immune cells, which can obscure the correlation between T cell filtration and PI3K pathway activation in tumor cells. What’s more, we observed similar changes on T cell infiltration in tumors with genomic gain in PIK3CA/PIK3CB as that in tumors with PTEN loss, which support the hypothesis that PI3K activation and PTEN loss yield similar impact on tumor immune microenvironment. All these findings indicated that the immune modulating effect of PTEN is at least partially attributed to PI3K pathway alteration, although some non-canonical functions of PTEN might also play a role amidst. As indicated in our data, PTEN loss is not synonymous with PI3K-Akt-mTOR activation, which is also revealed in published literature [42]. Except for the typical downregulation of PI3K-Akt-mTOR pathway, PTEN also exerts a series of non-canonical functions in a enzymatic or non-enzymatic manner [27, 35, 43, 44]. Emerging studies revealed that PTEN loss in prostate cancer resulted in an immunosuppressive tumor microenvironment through the activation of the Janus kinase 2 (JAK2)–signal transducer and activator of transcription 3 (STAT3) pathway and the subsequent secretion of immunosuppressive chemokines [38]. There are also studies reported that PTEN loss induced secretion of immunosuppressive cytokines via the activation of NF-κB pathway [37, 45]. The exact pathway by with PTEN exerts the immune modulating function is yet to be verified by further studies.

In addition to PTEN loss, more and more oncogenic events had been associated with tumor intrinsic resistance to immunotherapy. WNT/b-catenin pathway activation is also constantly detected in tumor developing resistance to immunotherapy [30, 46]. A recent published study has proven the correlation between wnt/b-catenin pathway activation and immune exclusion across human cancers [47]. Also, gain-of-function mutations in FGFR3, as well as activation of PPAR-γ pathway, have been associated with the T cell exclusion in tumor microenvironment in bladder cancer [18]. EGFR mutation in lung cancer is a well-known oncogene that impairs response to immunotherapy, which is attributed to an uninflamed phenotype and weak immunotherapy [48–50]. Of course, the above-mentioned oncogenic events didn’t account for all the tumors with the non-T-cell-inflamed phenotype, the full picture of which warrants continued effort on further research.

We found that PTEN status was a robust predictor for response to immunotherapy including PD-1 blockade and CTLA-4 blockade, although it was not associated with prognosis among patients without receiving immunotherapy. Our findings demonstrate that PTEN functions at the interface between cancer and tumor microenvironment and can eventually alter therapeutic outcome of immunotherapy. In the era of immunotherapy and precision medicine, our findings will translate into the molecular approach to classify the subpopulation of cancer patients that have greater chance of responding to immunotherapy. Also, with the implication on the mechanism by which PTEN modulate the immune microenvironment, we might be able to develop strategy to manipulate immune landscape and augment the therapeutic efficacy of immunotherapy in the clinical practice. Inhibitors targeting PI3K-Akt-mTOR pathway had shown promising efficacy in improving immunotherapy response in selected cancer type [9, 41, 51]. However, considering the multifaceted function of PTEN in all kinds of biological process, further study is warranted to bring in-depth understanding into its role in immune microenvironment and immunotherapy before we can actually exploit these knowledge in clinical practice.

Even with the promising findings, limitations of the present study need to be clearly addressed. First of all, the current work was merely based on the in silico analysis of TCGA, with all the immune parameters inferred from the transcriptome data. All these findings need to be confirmed by further study integrating IHC data or protein data. Also, data regarding PTEN status or PI3K-Akt-mTOR alteration were not derived from pure tumor cells, but from the tumor mass containing tumor cells as well as a small subset of stromal cells, infiltrating immune cells, whose confounding effect cannot be ruled out. Lastly, our analysis regarding genomic alteration of PTEN is limited to point mutations, small insertions and deletions, as well as copy number variants, whereas other types of genomic changes such as gene fusions or large-scale structural variants remain to be explored.

## Conclusions

In conclusion, the present study is the first to explore the immunosuppressive effect of PTEN and its potential mechanism in a pan-cancer setting. This set of data sufficiently motivated further effort on unraveling the detailed mechanism of immune exclusion mediated by PTEN loss, and developing immune modulating strategy to augment immunotherapy.

## Supplementary Information

**Additional file 1: Supplementary Table 1**. Summary of the basic information of 21 solid tumors. **Supplementary Table 2**. Detailed information of Chen et al. cohort. **Supplementary Table 3**. Detailed information of Riaz et al. cohort

**Additional file 2: Supplementary Figure 1**. Association of PTEN genomic alteration with PTEN expression and key pathway activation. Difference of PTEN mRNA expression, PI3K-AKT-mTOR pathway activation(represented by p-AKT [phosphorylated at Thr308 or Ser473], p-mTOR [phosphorylated at Ser2448], and ssGSEA score of PI3K pathway) and STAT3 activation (p-STAT3 [phosphorylated at Tyr705] between tumors with genomic PTEN loss and tumors with genomic Intact PTEN for each tumor types. *p < 0.05; **p < 0.01; ***p < 0.001; ****p < 0.0001; ns, not significant. **Supplementary Figure 2**. Gene set enrichment analysis (GSEA) of T-cell inflamed signature between tumors with PTEN loss and tumors with Intact PTEN. T-cell inflamed signature was highly enriched in tumors with intact PTEN for multiple cancer types; but for a few other cancer types, T-cell inflamed signature was highly enriched in tumors with PTEN loss. **Supplementary Figure 3**. Correlation of innate immune population with genomic alteration in PTEN, PIK3CA, PIK3CB. (a) Difference in the infiltration of innate immune cells, including dendritic cells (DC), macrophages and natural killer cells (NK) between tumor with PTEN loss and tumor with Intact PTEN; (b) Difference in the infiltration of innate immune cells (DC, macrophages and NK) between tumor with PIK3CA loss and tumor with wide-type PIK3CA; (c) Difference in the infiltration of innate immune cells (DC, macrophages and NK) between tumor with PIK3CB loss and tumor with wide-type PIK3CB. *p < 0.05; **p < 0.01; ***p < 0.001; ****p < 0.0001; ns, not significant. **Supplementary Figure 4**. Association of PTEN genomic alteration with expression of immunosuppressive markers. Logarithmic transferred mRNA expression of immunosuppressive genes (FOXP3, IDO1, CCL2, CSF1 and IL6) that had been reported to be associated with PTEN were compared between tumors with genomic PTEN loss and tumors with genomic Intact PTEN for each tumor types. *p < 0.05; **p < 0.01; ***p < 0.001; ****p < 0.0001; ns, not significant. **Supplementary Figure 5**. Survival impact of PTEN in Pan-cancer. Kaplan-Meier plots of overall survival difference between tumors with PTEN loss and tumors with Intact PTEN for each tumor type

## Data Availability

All the data applied in the present study were obtained from the publicly available databases: cBioportal database (https://www.cbioportal.org/); GEO database (GSE62944 https://www.ncbi.nlm.nih.gov/geo/query/acc.cgi?acc=GSE62944); TCPA database (https://tcpaportal.org/tcpa/my_protein.html).

## Abbreviations

BLCA: Bladder Urothelial Carcinoma
BRCA: Breast invasive carcinoma
COAD: Colon adenocarcinoma
GBM: Glioblastoma multiforme
HNSC: Head and Neck squamous cell carcinoma
KICH: Kidney Chromophobe
KIRC: Kidney renal clear cell carcinoma
KIRP: Kidney renal papillary cell carcinoma
LGG: Brain Lower Grade Glioma
LIHC: Liver hepatocellular carcinoma
LUAD: Lung adenocarcinoma
LUSC: Lung squamous cell carcinoma
OV: Ovarian serous cystadenocarcinoma
PAAD: Pancreatic adenocarcinoma
PRAD: Prostate adenocarcinoma
READ: Rectum adenocarcinoma
SARC: Sarcoma
SKCM: Skin Cutaneous Melanoma
STAD: Stomach adenocarcinoma
THCA: Thyroid carcinoma
UCEC: Uterine Corpus Endometrial Carcinoma
Th1: Type 1 T helper cells
Th2: Type 2 T helper cells
Tem: Effector memory T cells
Tcm: Central memory T cells
TFH: Follicular T helper cells
TReg: Regulatory T cells
Tgd: γδ T cells
NK cells: Nature killer cells
DC: Dendritic cells
aDC: activated dendritic cell
pDC: plasmacytoid dendritic cell
iDC: immature dendritic cell
