# Supplementary figures and images for: PTEN loss correlates with T cell exclusion across human cancers
Source: BMC Cancer. 2021 Apr 19;21:429. doi: 10.1186/s12885-021-08114-x (PMC8054401; doi:10.1186/s12885-021-08114-x)

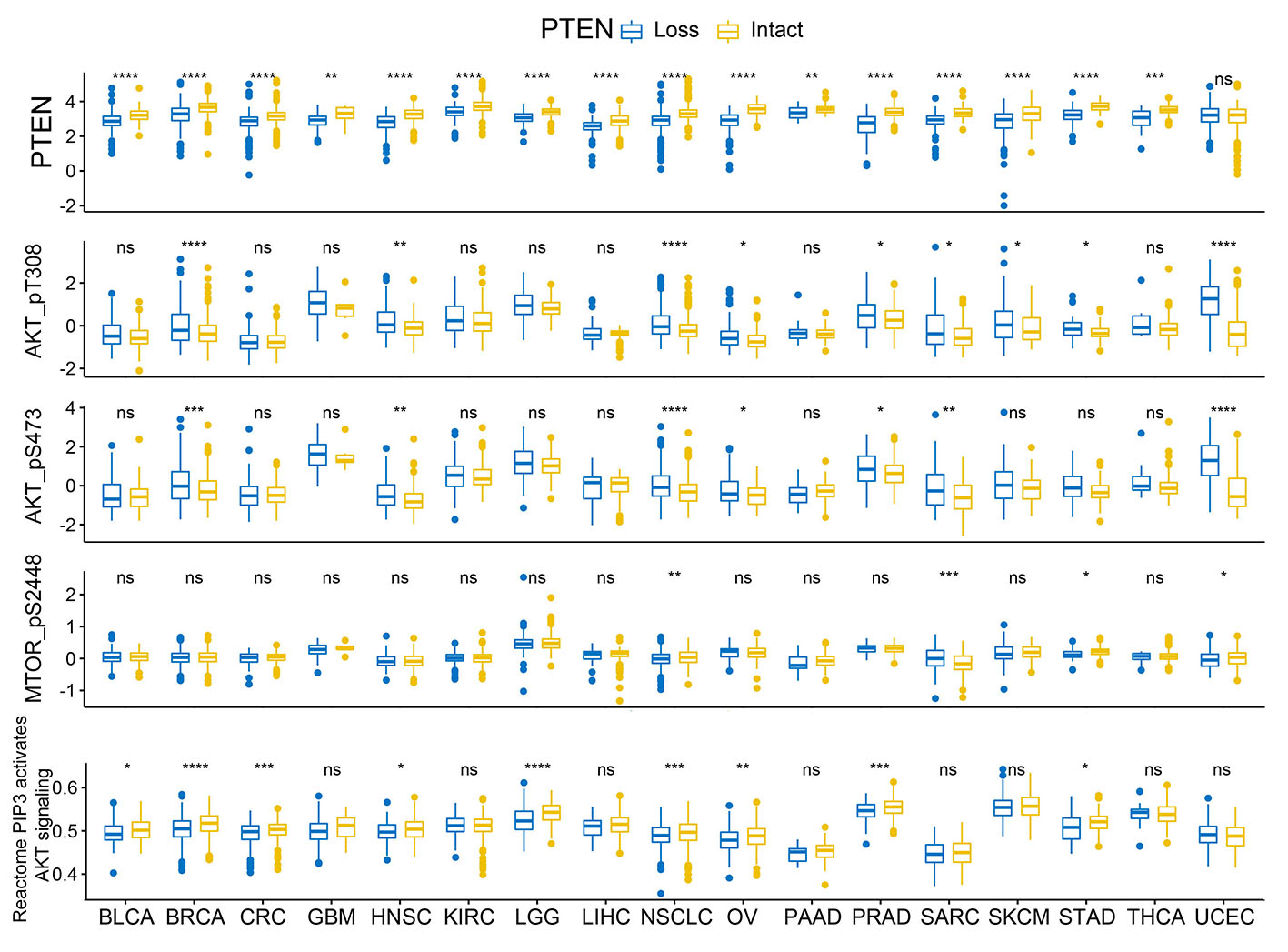

Supplement: Supplementary file 2 — Additional file 2: Supplementary Figure 1. Association of PTEN genomic alteration with PTEN expression and key pathway activation. Difference of PTEN mRNA expression, PI3K-AKT-mTOR pathway activation(represented by p-AKT [phosphorylated at Thr308 or Ser473], p-mTOR [phosphorylated at Ser2448], and ssGSEA score of PI3K pathway) and STAT3 activation (p-STAT3 [phosphorylated at Tyr705] between tumors with genomic PTEN loss and tumors with genomic Intact PTEN for each tumor types. *p < 0.05; **p < 0.01; ***p < 0.001; ****p < 0.0001; ns, not significant. Supplementary Figure 2. Gene set enrichment analysis (GSEA) of T-cell inflamed signature between tumors with PTEN loss and tumors with Intact PTEN. T-cell inflamed signature was highly enriched in tumors with intact PTEN for multiple cancer types; but for a few other cancer types, T-cell inflamed signature was highly enriched in tumors with PTEN loss. Supplementary Figure 3. Correlation of innate immune population with genomic alteration in PTEN, PIK3CA, PIK3CB. (a) Difference in the infiltration of innate immune cells, including dendritic cells (DC), macrophages and natural killer cells (NK) between tumor with PTEN loss and tumor with Intact PTEN; (b) Difference in the infiltration of innate immune cells (DC, macrophages and NK) between tumor with PIK3CA loss and tumor with wide-type PIK3CA; (c) Difference in the infiltration of innate immune cells (DC, macrophages and NK) between tumor with PIK3CB loss and tumor with wide-type PIK3CB. *p < 0.05; **p < 0.01; ***p < 0.001; ****p < 0.0001; ns, not significant. Supplementary Figure 4. Association of PTEN genomic alteration with expression of immunosuppressive markers. Logarithmic transferred mRNA expression of immunosuppressive genes (FOXP3, IDO1, CCL2, CSF1 and IL6) that had been reported to be associated with PTEN were compared between tumors with genomic PTEN loss and tumors with genomic Intact PTEN for each tumor types. *p < 0.05; **p < 0.01; ***p < 0.001; ****p < 0.00 [file 12885_2021_8114_MOESM2_ESM.zip › sFig 1R2.jpg]

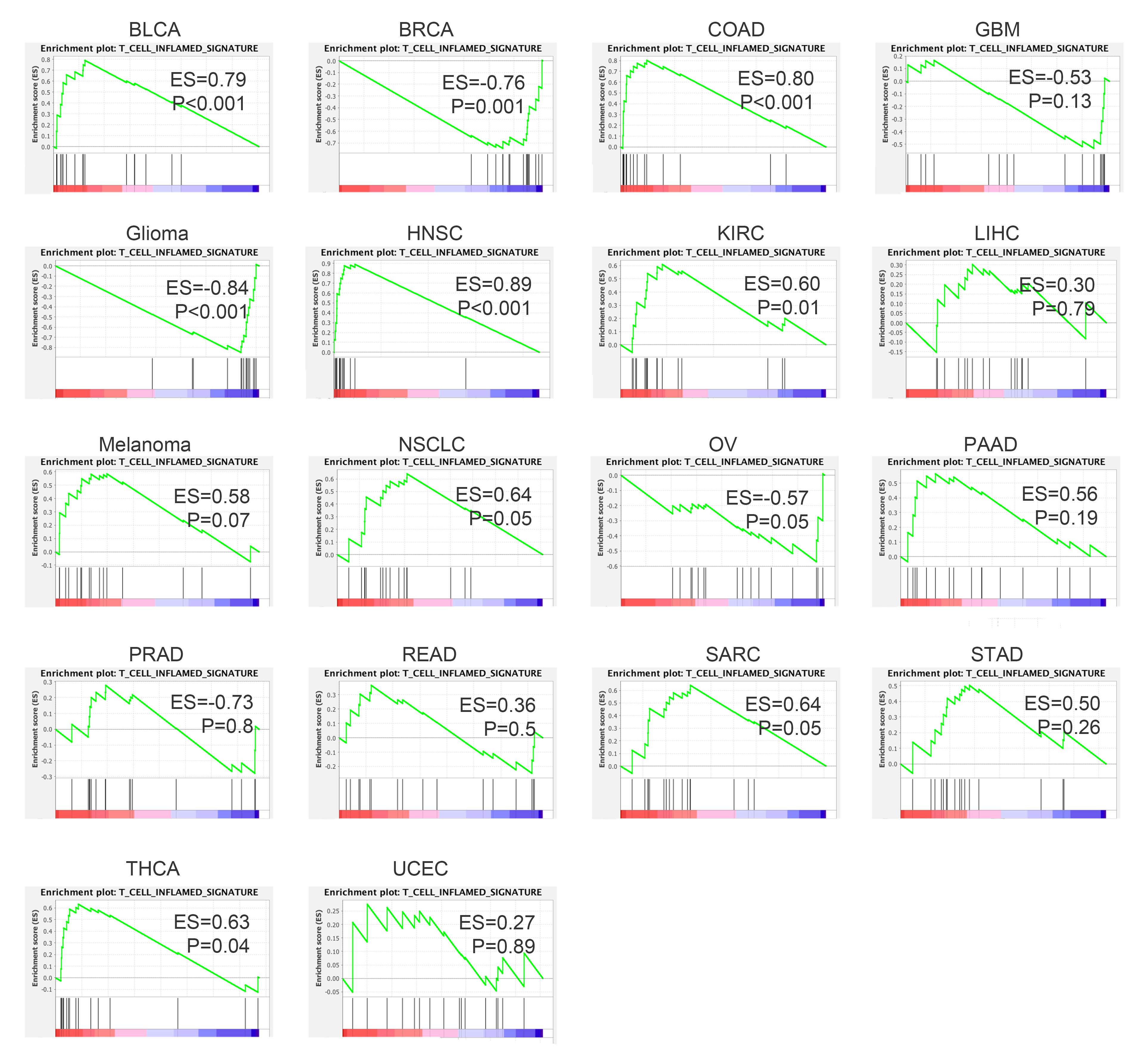

Supplement: Supplementary file 2 — Additional file 2: Supplementary Figure 1. Association of PTEN genomic alteration with PTEN expression and key pathway activation. Difference of PTEN mRNA expression, PI3K-AKT-mTOR pathway activation(represented by p-AKT [phosphorylated at Thr308 or Ser473], p-mTOR [phosphorylated at Ser2448], and ssGSEA score of PI3K pathway) and STAT3 activation (p-STAT3 [phosphorylated at Tyr705] between tumors with genomic PTEN loss and tumors with genomic Intact PTEN for each tumor types. *p < 0.05; **p < 0.01; ***p < 0.001; ****p < 0.0001; ns, not significant. Supplementary Figure 2. Gene set enrichment analysis (GSEA) of T-cell inflamed signature between tumors with PTEN loss and tumors with Intact PTEN. T-cell inflamed signature was highly enriched in tumors with intact PTEN for multiple cancer types; but for a few other cancer types, T-cell inflamed signature was highly enriched in tumors with PTEN loss. Supplementary Figure 3. Correlation of innate immune population with genomic alteration in PTEN, PIK3CA, PIK3CB. (a) Difference in the infiltration of innate immune cells, including dendritic cells (DC), macrophages and natural killer cells (NK) between tumor with PTEN loss and tumor with Intact PTEN; (b) Difference in the infiltration of innate immune cells (DC, macrophages and NK) between tumor with PIK3CA loss and tumor with wide-type PIK3CA; (c) Difference in the infiltration of innate immune cells (DC, macrophages and NK) between tumor with PIK3CB loss and tumor with wide-type PIK3CB. *p < 0.05; **p < 0.01; ***p < 0.001; ****p < 0.0001; ns, not significant. Supplementary Figure 4. Association of PTEN genomic alteration with expression of immunosuppressive markers. Logarithmic transferred mRNA expression of immunosuppressive genes (FOXP3, IDO1, CCL2, CSF1 and IL6) that had been reported to be associated with PTEN were compared between tumors with genomic PTEN loss and tumors with genomic Intact PTEN for each tumor types. *p < 0.05; **p < 0.01; ***p < 0.001; ****p < 0.00 [file 12885_2021_8114_MOESM2_ESM.zip › sFig 2R2.jpg]

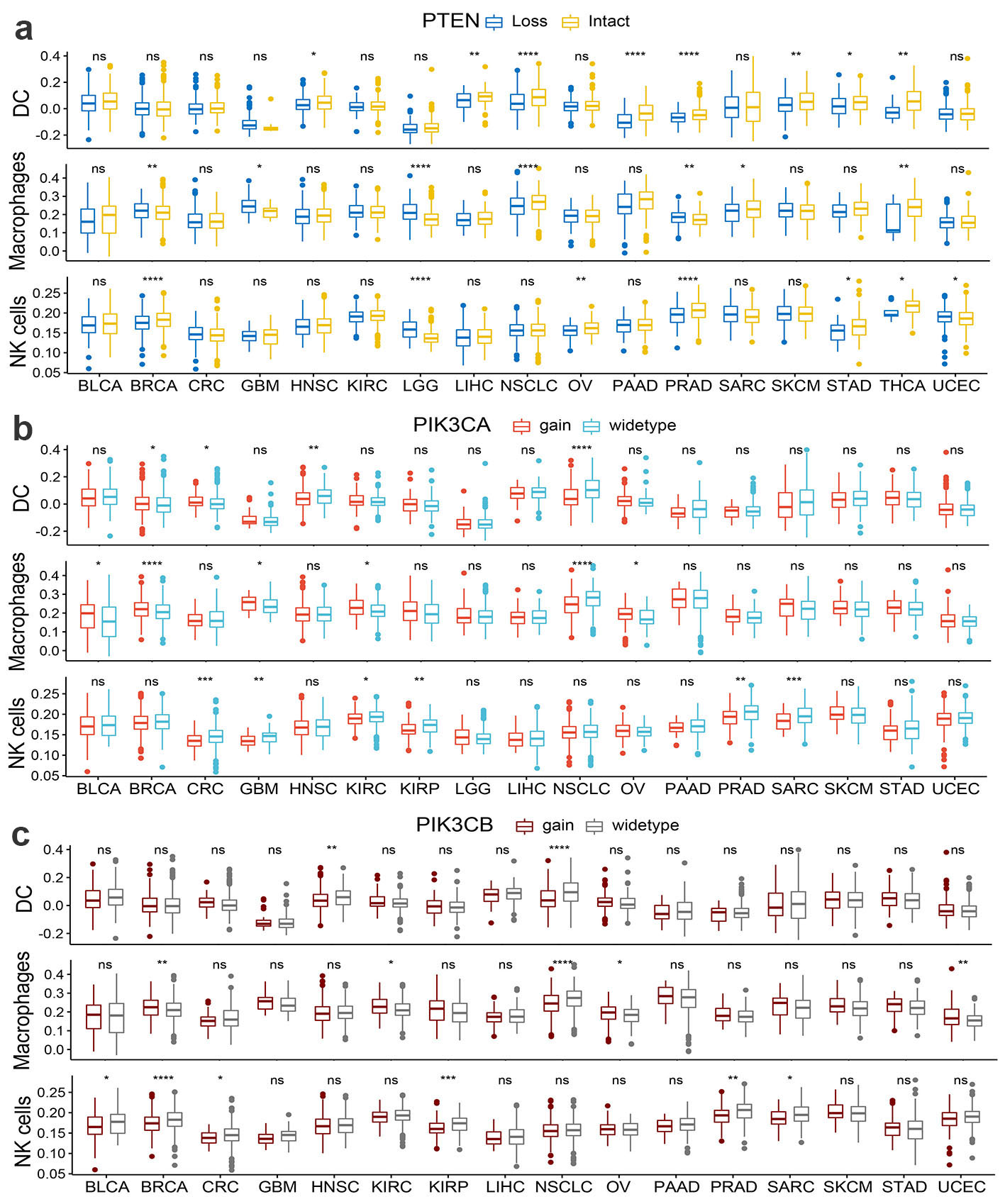

Supplement: Supplementary file 2 — Additional file 2: Supplementary Figure 1. Association of PTEN genomic alteration with PTEN expression and key pathway activation. Difference of PTEN mRNA expression, PI3K-AKT-mTOR pathway activation(represented by p-AKT [phosphorylated at Thr308 or Ser473], p-mTOR [phosphorylated at Ser2448], and ssGSEA score of PI3K pathway) and STAT3 activation (p-STAT3 [phosphorylated at Tyr705] between tumors with genomic PTEN loss and tumors with genomic Intact PTEN for each tumor types. *p < 0.05; **p < 0.01; ***p < 0.001; ****p < 0.0001; ns, not significant. Supplementary Figure 2. Gene set enrichment analysis (GSEA) of T-cell inflamed signature between tumors with PTEN loss and tumors with Intact PTEN. T-cell inflamed signature was highly enriched in tumors with intact PTEN for multiple cancer types; but for a few other cancer types, T-cell inflamed signature was highly enriched in tumors with PTEN loss. Supplementary Figure 3. Correlation of innate immune population with genomic alteration in PTEN, PIK3CA, PIK3CB. (a) Difference in the infiltration of innate immune cells, including dendritic cells (DC), macrophages and natural killer cells (NK) between tumor with PTEN loss and tumor with Intact PTEN; (b) Difference in the infiltration of innate immune cells (DC, macrophages and NK) between tumor with PIK3CA loss and tumor with wide-type PIK3CA; (c) Difference in the infiltration of innate immune cells (DC, macrophages and NK) between tumor with PIK3CB loss and tumor with wide-type PIK3CB. *p < 0.05; **p < 0.01; ***p < 0.001; ****p < 0.0001; ns, not significant. Supplementary Figure 4. Association of PTEN genomic alteration with expression of immunosuppressive markers. Logarithmic transferred mRNA expression of immunosuppressive genes (FOXP3, IDO1, CCL2, CSF1 and IL6) that had been reported to be associated with PTEN were compared between tumors with genomic PTEN loss and tumors with genomic Intact PTEN for each tumor types. *p < 0.05; **p < 0.01; ***p < 0.001; ****p < 0.00 [file 12885_2021_8114_MOESM2_ESM.zip › sFig 3R2.jpg]

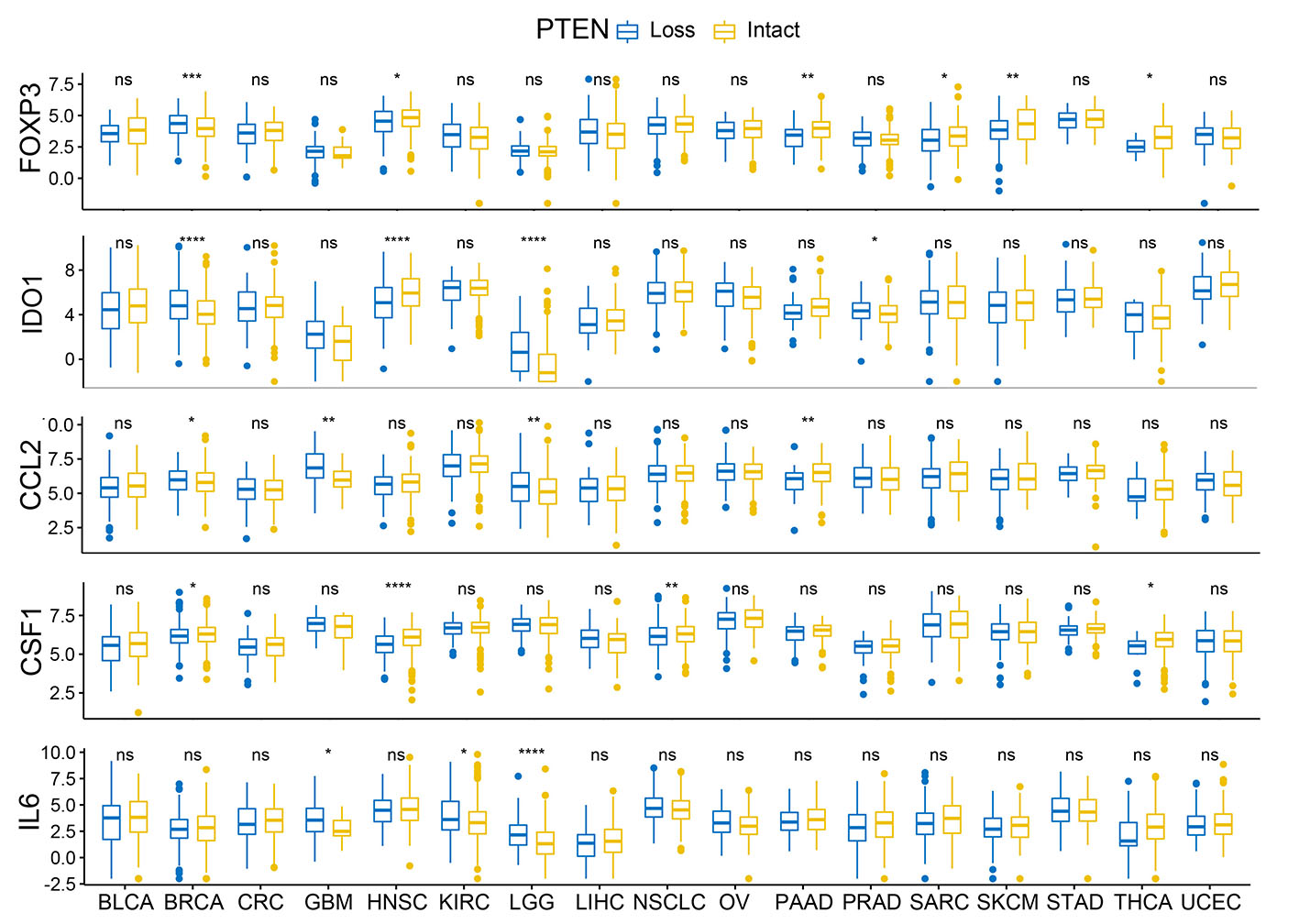

Supplement: Supplementary file 2 — Additional file 2: Supplementary Figure 1. Association of PTEN genomic alteration with PTEN expression and key pathway activation. Difference of PTEN mRNA expression, PI3K-AKT-mTOR pathway activation(represented by p-AKT [phosphorylated at Thr308 or Ser473], p-mTOR [phosphorylated at Ser2448], and ssGSEA score of PI3K pathway) and STAT3 activation (p-STAT3 [phosphorylated at Tyr705] between tumors with genomic PTEN loss and tumors with genomic Intact PTEN for each tumor types. *p < 0.05; **p < 0.01; ***p < 0.001; ****p < 0.0001; ns, not significant. Supplementary Figure 2. Gene set enrichment analysis (GSEA) of T-cell inflamed signature between tumors with PTEN loss and tumors with Intact PTEN. T-cell inflamed signature was highly enriched in tumors with intact PTEN for multiple cancer types; but for a few other cancer types, T-cell inflamed signature was highly enriched in tumors with PTEN loss. Supplementary Figure 3. Correlation of innate immune population with genomic alteration in PTEN, PIK3CA, PIK3CB. (a) Difference in the infiltration of innate immune cells, including dendritic cells (DC), macrophages and natural killer cells (NK) between tumor with PTEN loss and tumor with Intact PTEN; (b) Difference in the infiltration of innate immune cells (DC, macrophages and NK) between tumor with PIK3CA loss and tumor with wide-type PIK3CA; (c) Difference in the infiltration of innate immune cells (DC, macrophages and NK) between tumor with PIK3CB loss and tumor with wide-type PIK3CB. *p < 0.05; **p < 0.01; ***p < 0.001; ****p < 0.0001; ns, not significant. Supplementary Figure 4. Association of PTEN genomic alteration with expression of immunosuppressive markers. Logarithmic transferred mRNA expression of immunosuppressive genes (FOXP3, IDO1, CCL2, CSF1 and IL6) that had been reported to be associated with PTEN were compared between tumors with genomic PTEN loss and tumors with genomic Intact PTEN for each tumor types. *p < 0.05; **p < 0.01; ***p < 0.001; ****p < 0.00 [file 12885_2021_8114_MOESM2_ESM.zip › sFig 4R2.jpg]

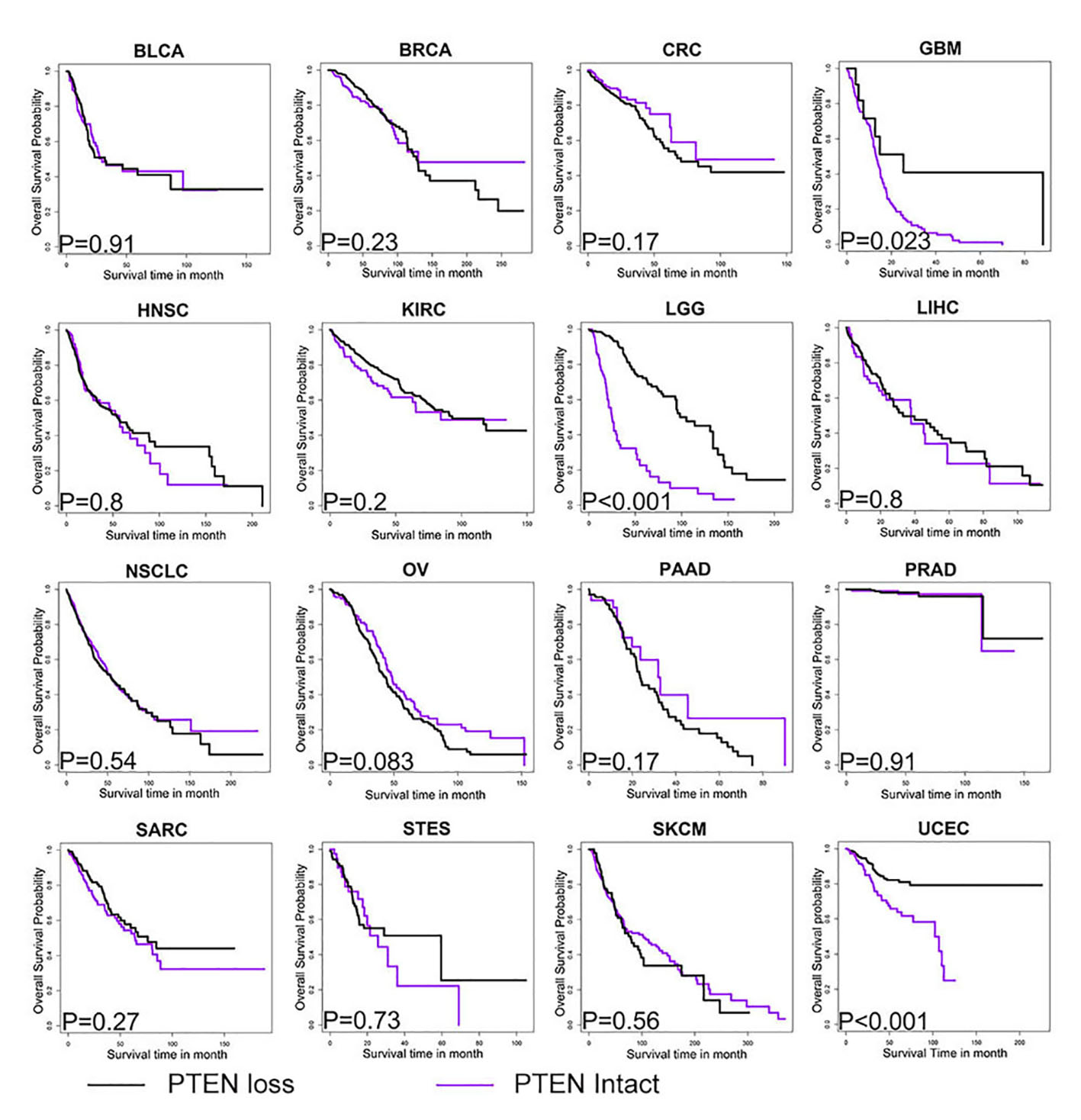

Supplement: Supplementary file 2 — Additional file 2: Supplementary Figure 1. Association of PTEN genomic alteration with PTEN expression and key pathway activation. Difference of PTEN mRNA expression, PI3K-AKT-mTOR pathway activation(represented by p-AKT [phosphorylated at Thr308 or Ser473], p-mTOR [phosphorylated at Ser2448], and ssGSEA score of PI3K pathway) and STAT3 activation (p-STAT3 [phosphorylated at Tyr705] between tumors with genomic PTEN loss and tumors with genomic Intact PTEN for each tumor types. *p < 0.05; **p < 0.01; ***p < 0.001; ****p < 0.0001; ns, not significant. Supplementary Figure 2. Gene set enrichment analysis (GSEA) of T-cell inflamed signature between tumors with PTEN loss and tumors with Intact PTEN. T-cell inflamed signature was highly enriched in tumors with intact PTEN for multiple cancer types; but for a few other cancer types, T-cell inflamed signature was highly enriched in tumors with PTEN loss. Supplementary Figure 3. Correlation of innate immune population with genomic alteration in PTEN, PIK3CA, PIK3CB. (a) Difference in the infiltration of innate immune cells, including dendritic cells (DC), macrophages and natural killer cells (NK) between tumor with PTEN loss and tumor with Intact PTEN; (b) Difference in the infiltration of innate immune cells (DC, macrophages and NK) between tumor with PIK3CA loss and tumor with wide-type PIK3CA; (c) Difference in the infiltration of innate immune cells (DC, macrophages and NK) between tumor with PIK3CB loss and tumor with wide-type PIK3CB. *p < 0.05; **p < 0.01; ***p < 0.001; ****p < 0.0001; ns, not significant. Supplementary Figure 4. Association of PTEN genomic alteration with expression of immunosuppressive markers. Logarithmic transferred mRNA expression of immunosuppressive genes (FOXP3, IDO1, CCL2, CSF1 and IL6) that had been reported to be associated with PTEN were compared between tumors with genomic PTEN loss and tumors with genomic Intact PTEN for each tumor types. *p < 0.05; **p < 0.01; ***p < 0.001; ****p < 0.00 [file 12885_2021_8114_MOESM2_ESM.zip › sfig 5R2.jpg]
